# Neuroprotective effects of omega‐3 oil on aluminium‐induced oxidative stress in the hippocampus of female Wistar rats

**DOI:** 10.1002/alz.085771

**Published:** 2025-01-09

**Authors:** Tangban Cynthia Nyen, Ajibade Testimony Prescillia

**Affiliations:** ^1^ University of Ibadan, Ibadan, Oyo Nigeria

## Abstract

**Background:**

Aluminium chloride, an environmental toxicant induces neurotoxicity by increasing anxiety, causing cognitive deficit and memory impairment due to its effects on the hippocampus. Omega‐3 oil has been shown to improve cognition in neurologic disorders.

**Method:**

Forty adult female rats were divided into 4 groups (n = 10). Group A received distilled water, group B received 100mg/kg aluminium chloride_,_ group C received 300mg/kg of omega‐3 oil and group D received 100mg/kg Alcl_3_ and 300mg/kg omega‐3 one hour interval. All treatment was done orally for 28 days. Neurobehavioural test was done and the rats sacrificed on day 29. The hippocampus was dissected out and some preserved in phosphate buffered saline at 4°C and pH, 7.2 for oxidative stress and antioxidant markers, while others fixed in 10% formol‐saline for histological and immunohistochemical studies. Data analyzed using one way analysis of variance at p<0.05.

**Result:**

The Alcl_3_‐treated rats showed deficit in cognition and spatial memory, increased oxidative stress marker and decreased antioxidant system compared with the control and omega‐3 oil‐treated groups. The hippocampus showed Pyramidal cell degeneration, vacuolation, karyorrhexis and pyknosis, and increased reactive astrocyte and B‐Cell lymphoma‐associated X‐protein (BAX) in the Alcl_3_‐treated group compared with the control and omega‐3 oil‐treated groups. Aluminium chloride induced oxidative stress, negative cognitive behaviour, memory loss and decreased the antioxidant capacity in the hippocampus of Wistar rats

**Conclusion:**

Omega‐3 oil attenuated these negative effects suggesting its neuroprotective capability possibly through upregulating the antioxidant system, reducing astrogliosis or by preventing the pro‐apoptotic cascade.

